# Coordinating a new approach to basic research into Parkinson’s disease

**DOI:** 10.7554/eLife.51167

**Published:** 2019-09-25

**Authors:** Randy Schekman, Ekemini AU Riley

**Affiliations:** 1 Department of Molecular and Cell BiologyUniversity of California BerkeleyBerkeleyUnited States; 2 Milken Institute Center for Strategic PhilanthropyWashingtonUnited States

**Keywords:** Parkinson's disease, neurodegenerative disorders, open science, philanthropy, team science, organization of research, None

## Abstract

The Aligning Science Across Parkinson’s (ASAP) initiative is building an international network of researchers to improve our understanding of the biology underlying Parkinson's disease. Developing a better understanding of how the disease originates and progresses will, we hope, lead to new therapies. The ASAP initiative will incentivize collaboration between the existing PD research community and other researchers and will be committed to open-science practices.

## Introduction

Parkinson’s disease is a progressive neurodegenerative disorder characterized primarily by debilitating motor symptoms, as well as gastrointestinal distress and impaired sleep and cognition. More than six million people are known to be living with the disease, and it is the fastest growing neurodegenerative disorder worldwide (GBD 2016 Parkinson's Disease Collaborators, 2018). Like Alzheimer’s disease, Parkinson's disease (PD) is usually a disease of old age; however, many patients develop symptoms of the disease in their fourth or fifth decade – the very years that should be the prime of their lives. In the 200 years since PD was described, and despite recent decades of intense clinical work, only a few therapies have been developed to mitigate some aspects of the movement disorder. Nothing has helped to arrest or slow the progression of the disease, though not for lack of searching.

One problem is that funding for PD research is modest at best: in the United States, for example, the economic burden of PD is estimated to be $52bn per year, yet the National Institutes of Health (NIH) only spent an estimated $168 m on PD research in FY 2017 (which was just 6.5% of what it spent on neurodegenerative diseases). And although two non-profit organizations – the Michael J. Fox Foundation for Parkinson’s Research and the Parkinson’s Foundation – spent a further $108 m on PD research in FY 2017, it is estimated that 1.04 million Americans have PD, so total spending on research is about $265 per patient, which is a tiny fraction of the cost borne by the families who care for their loved ones as the disease takes its course.

The PD landscape has shifted significantly since the first genetic link to the disease was discovered in 1997. Since then, the field has experienced a revolution in our understanding of genetic contributors to the disease, which has spurred industry involvement. Among the proteins implicated by genetic studies, alpha-synuclein has been linked to PD through it presence in the Lewy bodies that are found in the substantia nigra of patients who succumb to the disease (Spillantini et al., 1998). Other genetic risk factors that have emerged from sequencing efforts include the *GBA* gene, which encodes a lysosomal enzyme, glucocerebrosidase; *LRRK2*, a protein kinase; and *PINK1* and *PARKIN*, two genes linked to the turnover of mitochondria (reviewed in Klein and Westenberger, 2012; Billingsley et al., 2018).

Yet, even with these leads, the functional connection to the more common idiopathic form of the disease is not known. More recently we have come to recognize that the disease process may begin sometimes decades before noticeable symptoms appear, raising the possibility that early treatment might eventually prevent the most devastating aspects of PD (Postuma and Berg, 2016). There is also a growing appreciation for the involvement of non-neuronal cell types, the immune system, and peripheral organ systems. If we have learned anything so far, it is that PD is a multifaceted and complex disease, more like a spectrum disorder, that involves multiple biological systems and is triggered by both genetic and environmental factors.

Because we do not fully understand the onset and progression of PD at the molecular and cellular level, our ability to develop quantitative diagnostic assays and expand therapeutic options is hampered. Treatment has not advanced substantially since the advent of levodopa more than 40 years ago, notwithstanding the more recent invention of deep brain stimulation. Moreover, we still do not know the answers to fundamental questions about initiating factors and events, genetic risk, selective neuronal death, compensatory pathways, neuro-immune mediators, and much more.

Aligning Science Across Parkinson’s (ASAP) was conceived as a vehicle to help answer these questions. Our goal is to fund a basic research program that amplifies and coordinates the efforts of researchers around the world, both inside and outside of the existing PD community. While much valuable research is underway, an additional coordinated push, specifically on basic disease mechanisms, may yield the breakthroughs needed to develop therapies that are more than palliative and go to the core of the disease. Harnessing the talents of collaborative teams employing various techniques (such as high-resolution microscopy, single cell technologies, advanced data analysis, and stem cell and human brain organoid culture) will be critical if we are to achieve this.

Launched two years ago as a joint project of the Milken Institute Center for Strategic Philanthropy and the Sergey Brin Family Foundation, ASAP will allocate financial resources that significantly enlarge the basic science effort currently supported by the government and private foundations with the goal of understanding the underlying biology of PD. An international call for pre-proposals will be announced in October of this year. Details will be available on the ASAP website.

## Planning and objectives

In 2017, we began an intensive planning process to identify research areas that had the potential to transform our understanding of the biology underlying PD, and to consider the resources that would be needed to enable this research. We engaged more than 100 PD and non-PD scientists from around the world with expertise in a range of disciplines including cell biology, genetics, immunology, microbiology, and neuromodulation. We further sought perspectives from other sectors including non-profit organizations, government, industry, and patient advocates.

The planning process revealed several perceived deficiencies and limiting factors in PD research. Investigators cited risk aversion to novel concepts, often resulting in a lack of robust funding to test new ideas. Further, a lack of scale in areas such as investment, cohort size, and breadth of scientific expertise was seen as contributing to underpowered studies unable to distinguish weak signals from the considerable noise of a diverse and multifaceted disease. This is where philanthropy can play a major role: philanthropic capital has the ability to shoulder risk and support studies that might not otherwise be funded, and to encourage a more collaborative and open approach to research.

**Figure 1.**
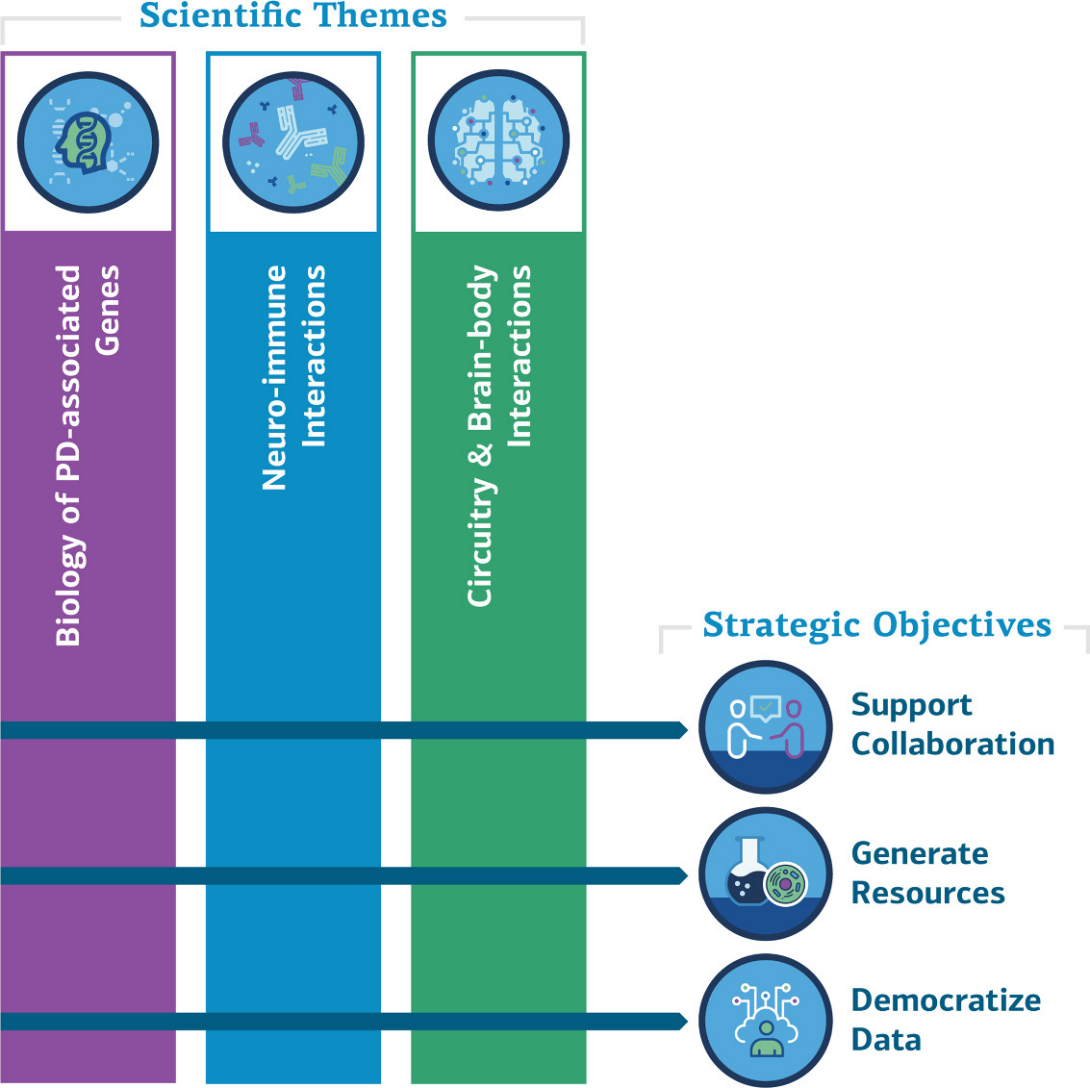
ASAP scientific themes and strategic objectives. The ASAP initiative will support collaboration between the existing PD research community and other researchers, generate resources for research, and make data widely available. It will fund research in the three themes shown above, as well as research on how these themes influence the progression of PD.

As a result of this planning process we have developed a research roadmap that focuses on three objectives (see below; Figure 1) and has four research themes (see next section).

### Objective 1: Supporting meaningful collaboration

For almost a century curiosity-driven basic science – mostly on projects initiated by individual investigators – has been the engine of advance in biomedicine. And yet, for certain difficult problems that require a variety of skills to tackle, a team effort may be preferable. This approach is made more difficult in academic science today by a reward structure that favors the individual over the team. Research laboratories, led by a single faculty member, compete for attention as measured too often by first-author and senior-author positions on papers published in so-called high-profile journals. This has led to a decline in both the rigor of scientific research and its creative output (Alberts et al., 2014). Meanwhile, it is increasingly recognized that certain problems can only be solved if researchers from different disciplines work together. This has resulted in a growing body of literature on successful collaboration, particularly in sectors such as biotech, where team effort moves specific goals forward.

In academia, collaborations between groups are encouraged, and are sometimes even formed on the basis of an application for research funding, but these may at best result in one or two publications and are seldom enduring. Young scholars who spend a major fraction of their effort in such collaborations may actually be punished as review committees feel they are unable to assess the individual contribution of the early-career candidate under consideration for a grant or promotion. And yet, for the more infrequent long-term collaborations in which groups of diverse interest or skill do come together to tackle a difficult problem (such as the Human Genome Project), the payoff can greatly exceed the disparate efforts of unaffiliated laboratories.

What are the measures of a successful collaboration? How can we go about incentivizing such a program for ASAP? What tools are needed? And how will we identify the best people who are able to engage in a constructive collaboration of the scale we are proposing? In addition to studies on the ‘science of team science’, there are emerging experiments by funders, institutions, and publishers that can serve as models (Vogel et al., 2013; Klein and Falk-Krzesinski, 2017). For instance, an investigator’s past experience in collaborative engagements may be the surest measure of future potential. Accordingly, ASAP is looking for individuals around the world with a proven track record of collaboration in the research areas outlined below. Ideally, these individuals will join forces with one or more past collaborators to form teams that combine researchers from different disciplines and/or backgrounds (e.g., academic and biotech/pharma) and are not limited to traditional avenues of PD research. To ensure that early-career investigators are meaningfully integrated into teams, we will require that each proposed team include at least one early-career investigator (within one to seven years of their first academic appointment) in a lead position in order to be eligible for funding.

Successful teams must also be open to the following: sharing new results on a virtual platform among the ASAP groups; posting manuscripts at the point of journal submission on a preprint forum such as bioRxiv; and publishing finished work in a journal that offers immediate open access with a Creative Commons CC-BY license, in clear alignment with Plan S principles. It is our firm belief that open access and research transparency will accelerate the pace of discovery; as such, ASAP will join like-minded research funders and charitable foundations that support the move to make full and immediate open access to research publications a reality by joining cOAlition S. We will also seek to open lines of communication to the public, especially to patient groups.

This effort will be administered in collaboration with the Michael J. Fox Foundation, with staff support from the Milken Institute and the financial backing of the Sergey Brin Family Foundation. Competitive pre-proposals and full proposals will be evaluated by ASAP leadership, scientific staff at collaborating PD non-profits, and peer reviewers with expertise in the relevant disciplines.

Successful teams will be selected on the basis of several criteria meant to evaluate past collaborative behaviors, standing within their fields and connectedness within the research community, an interdisciplinary approach to tackling one of our scientific themes, the engagement of early-career and non-PD investigators, and commitment to the open-science policies we plan to promote.

The research program will be managed to encourage cooperation and collaboration amongst investigators and funders alike, to reduce redundancy, and enable researchers to pursue their most ambitious ideas. We will award up to $9m per team over three years, with a possible extension to five years with additional funds, to eligible teams that bring truly diverse expertise to bear on fundamental aspects of PD disease mechanisms. We have no target number of teams to fund. Our decisions will be based entirely on the quality of the teams and of their proposals.

### Objective 2: Generating research resources

To date, no simple biomarker can conveniently be applied to patients, family members, or to those who present with hyposmia and REM sleep disorder, which have been observed to precede motor symptoms in PD. And even for the traditional morphologic hallmark of the disease, brain Lewy bodies, no non-invasive probe is available. Diagnosis is made by microscopic inspection of a tissue biopsy taken on the death of a patient. Patients and their family members must rely on cognitive and behavioral diagnosis; absent a window into underlying biological processes, the uncertain path of the disease precludes any long-term planning. Chemical, protein or cellular biomarkers could reveal distinct types of PD and allow quantitative assessment of their progression. At a deeper level, the identification of predictable molecular changes may lead to new approaches to define the origin and mechanism of progression of the disease.

To enable biomarker discovery, ASAP will fund the establishment of research-enabling tools and platforms (such as isogenic induced pluripotent stem cell lines and longitudinal patient cohorts) to fuel mechanistic studies using human samples. We will enlarge the effort to identify biomarkers that can quantitatively measure disease progression. Further, ASAP will support the ongoing search for new genetic determinants of PD in ethnically diverse populations to bolster the search for rational drug targets and refine our understanding of disease risk. An initial investment of $150 m over the next five years will kick-off this work. These efforts aim to serve the entire neuroscience community – they are not limited to groups funded by ASAP.

### Objective 3: Democratizing data

Large-scale data-sharing and analysis is frequently hampered by the difficulty of integrating complex datasets: as a result, the effort required to pool and standardize large datasets often overwhelms individual investigators. ASAP will address these issues by making data and other research objects discoverable, publicly available, and fully integrated into the PD data ecosystem.

We expect to generate enormous datasets through projects such as the evaluation of patient samples collected from PD and matched family-member cohorts, and an expanded search for gene loci linked to familial forms of the disease. We will support the deposition of all such data, subject to privacy considerations, on publicly accessible databases. And while we intend to engage biotech and pharma in our effort, all resources from ASAP must be subject to our open-sharing and open-access policies.

## Scientific themes

ASAP will focus on three scientific themes discussed below, with the influence of these processes on the progression of PD being an important fourth theme.

### Theme 1: Biology of PD-associated genes

Genome-wide association studies have identified nearly 90 risk loci for PD, perhaps 20 of which are considered causal (Nalls, 2019). With current estimates attributing up to 30% of PD risk to genetic influences, a great deal of interest has developed around the functional significance of genes such as *SNCA, GBA, LRRK2*, *PINK1,* and *PARKIN* in normal cellular processes with obvious relevance to PD. What about the many other genes that are now being uncovered? Do they fit into a single neat pathway or, more likely, which pathways intervene to produce the spectrum of PD pathology? This molecular effort must be extended to brain circuits functionally linked to dopaminergic neurons in order to place the influences of these gene products in a physiologic context. Which experimental systems will best characterize the roles of these gene products: biochemical, cellular, brain organoid or animal models? Are specific genetic influences involved in the progression of roughly 30% of PD patients to dementia?

### Theme 2: Neuro-immune interactions

Neuroinflammation has long been implicated in PD, however the underlying molecular mechanisms mediating this process remain unknown (McGeer et al., 1988). In Alzheimer’s disease, this development has enabled genetic studies that implicate microglial cells, a primary component of the brain’s innate immune system, in the inflammatory response to neurodegeneration (Schapansky et al., 2015). Could these cells be responsible for clearing Lewy bodies or could extrinsic factors unleash an attack on dopaminergic neurons? Recent research suggests that PD is not purely a neurodegenerative disease of the brain, but involves peripheral organ systems including the immune system and microbiome.

### Theme 3: Circuitry and brain-body interactions

The recent understanding of PD as a multisystem disorder requires a more holistic approach that considers the interactions of various systems involved in the condition. This analysis must consider neuromodulatory dysfunction beyond dopamine, the substantia nigra, and other deep brain regions to include interactions across the entirety of the central and peripheral nervous systems. This will enable a better understanding of what potentiates motor and non-motor symptoms associated with the disease. It would be interesting to explore how new tools developed through the BRAIN Initiative could be integrated into basic PD research.

### Theme 4: Progression

It is increasingly recognized that by the time a patient is diagnosed, the pathophysiology of PD has been set in motion for years, possibly decades. The fact that 60–80% of dopamine-producing neurons in the substantia nigra have already degenerated upon diagnosis (Fearnley and Lees, 1991; Pakkenberg et al., 1991), and that non-motor precursor symptoms can be identified up to 20 years prior to that point, suggest that there may be opportunities to identify the presence of PD in early stages and intervene before the onset of symptoms. Yet, we know very little about how the brain becomes gradually compromised at the cellular level. This in turn limits our ability to identify biomarkers and develop mechanism-based diagnostic tools. This research area is interconnected with each of the aforementioned themes, as progression affects every aspect of the underlying disease pathology.

## Outlook

Ultimately, the vision for ASAP is to focus on the underlying biology of PD to elucidate novel pathways that will fuel the development of rational treatments. To be clear, the PD field has made significant strides, owing to the work of those before us, and we must take care to build on their efforts. We believe that a meaningful collaboration among internationally distributed laboratories, supported by substantial and sustained philanthropic resources, can make a difference above and beyond what has already been achieved.
